# Influence of general practitioners’ perceptions on social prescription of arts, nature, and physical activity for psychosocial health and well-being: a structural equation model approach

**DOI:** 10.3389/fpubh.2025.1649931

**Published:** 2025-12-10

**Authors:** Rashid Menhas, Muhammad Younas

**Affiliations:** 1 School of Nursing, Shandong Xiehe University, Jinan, China; 2 College of Humanities and Sciences, Prince Sultan University, Riyadh, Saudi Arabia

**Keywords:** social prescription, general practitioner, perception, arts, nature, physical activity

## Abstract

**Background:**

GPs are pivotal in promoting holistic health and well-being among patients, with the emerging concept of social prescription gaining attention. Understanding GPs’ knowledge, attitudes, and perceived influence regarding social prescription is crucial for practical integration into healthcare practice. Therefore, the current study addresses this research gap by comprehensively understanding the influence of general practitioners’ perceptions (knowledge, attitudes, and perceived effectiveness) on arts, nature, physical activity, and social prescription for psychosocial health and well-being.

**Methods:**

This research was exploratory, and a cross-sectional survey design was adopted to collect data at a single point in time from a sample of general practitioners in China. The primary data were gathered via a self-administered questionnaire survey. The data analysis for this inquiry used the Smart-PLS 3.2.9 software package.

**Results:**

A total of 701 general practitioners were recruited. The proposed hypotheses of the study are as follows: H1 (*t* = 10.418, *p* = 0.00); H2 (*t* = 2.772, *p* = 0.000); H3 (*t* = 0.172, *p* = 0.00); H4 (*t* = 79.05, *p* = 0.000); H5 (*t* = 1.272, *p* = 0.000); H6 (*t* = 0.655, *p* = 0.000); H7 (*t* = 0. 540, *p* = 0.000) H8 (*t* = 0.767, *p* = 0.000), H9 (*t* = 0.201, *p* = 0.000), H10 (*t* = 1.409, *p* = 0.000), and H11 (*t* = 0.395, *p* = 0.000) show significant relationships.

**Conclusion:**

People’s thoughts and behaviors related to social prescriptions from general practitioners involve a broad spectrum of factors, including deeming doctors more qualified and, therefore, more influential, with some beyond the doctor’s control. Among the key factors that can make or mar the success of social prescriptions, arts, nature, and physical activities strategies meant to boost health and well-being is the knowledge, attitudes, and beliefs of GPs that they are the contributing agents.

## Introduction

Social prescription programs are based on nonpharmacological interventions where primary care physicians recommend them to their patients for psychosocial health and well-being. Social prescription intervention innovations could help close health-related disparities and increase common health at the whole-community level (1). GPs act as social prescription sources of health and well-being by linking the extent to which people need help with services from the hospital and the community. The social prescribing movement emerged because of the recognition of the importance of the SDOH for patients’ health and well-being. Several patients may have nonmedical problems that worsen their overall health. GPs’ engagement in social prescribing confirms the primary public health agenda, as it is focused on well-timed prevention of diseases and improving the overall health condition related to the “health and well-being gap” (2). GPs occupy the most strategic location in diagnosing the social determinants of these conditions, as they usually occur during patient consultations. Active listening and comprehensive assessments can resolve these problems (3). However, the acceptability and feasibility of social prescribing by stakeholders are witnessed by medical doctors, healthcare professionals, and social service professionals (4).

A green prescription is recommended by the general practitioner, which has been proven to enhance the standard of living and the patient’s well-being. Moreover, significant evidence shows that GPs who actively participate in social prescribing interventions can increase patients’ psychological well-being and reduce their reliance on healthcare services, which comes from other stakeholders’ points of view, such as patients’ and GPs’ points of view (5). GPs prescribe various social prescriptions through social context and patient characteristics to promote psychosocial health and well-being (6, 7). GPs offer a comprehensive portfolio of activities aimed at patients’ well-being before resorting to medical intervention. Strengthening GPs’ knowledge, creating a positive attitude, and gaining an accepted opinion are three essential aspects that social prescribing should integrate into medical practice. The knowledge and understanding of GPs, attitudes, and rationale for their engagement with social prescribing are crucial for effectively implementing social prescribing. It is essential to understand not only the behavior and attitudes of GPs but also how GPs shape social prescribing practices and their participation and, therefore, influence the successful implementation of social prescribing projects (3, 4).

The demand for nonpharmacological interventions under social prescribing continues to rise for physical and mental health (8, 9). This suggests that many nature-based and green social prescriptions, such as gardens, nature walks, and nature conservation activities, are healthy and good for well-being (10). Some positive changes evidenced by policy interventions include the rejuvenation of physical activities, stress reduction, obesity prevention, and improved overall health and well-being through programs such as park prescriptions and outdoor walking groups (11). Participating in art shows is one of the most important benefits for older adults. Research has shown that the arts positively influence people’s health and well-being, including self-esteem, in the latter part of life (12). Moreover, social prescribing offers patients various nonmedical referrals for community arts events, walking clubs, and communal gardens to ensure that they feel the connection they need to maintain their psychosocial health (13).

## Literature review

### Theoretical framework

#### Normalization process theory (NPT)

**Figure 1:**
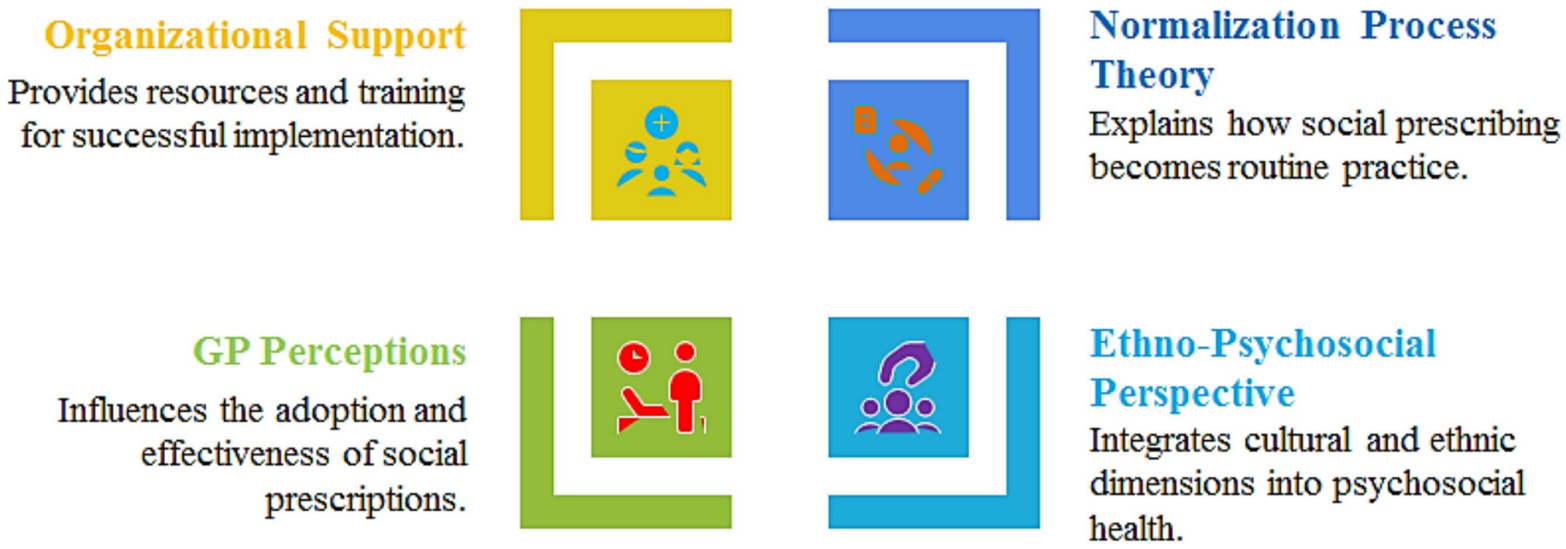
Theoretical framework.

Normalization process theory (NPT) is a theoretical framework that can potentially be utilized to explain aspects of implementing, embedding, and maintaining such interventions in service settings. Teamwork is vital in normalization process theory (NPT), as it explains what individuals and teams do to embed a new practice into their everyday routines and make it a habit (14). With respect to arts, nature, and physical activity social prescribing, the NPT could explain how GPs understand and engage with these practices and how their understanding may affect the things that help or make it difficult to embed the practices and, in turn, the outcomes (14). The concept of coherence underpins coherence, and it concerns how individuals or actors come to understand a new practice and how they understand the usefulness of the practice. NPT could explain how GPs understand and engage with arts, nature, and physical activity social prescribing and how their understanding may affect the things that help or make embedding practices and, in turn, outcomes difficult (15). Therefore, it is arguable that GPs are more or less likely to understand arts, nature, or physical activity social prescribing with patient health and well-being needs, depending on how coherent they think the practice is. Another component of the NPT is collective action, which includes the action-oriented work done together to embed a new practice (14). Arts on prescription, nature on prescription, and physical activity on prescription are currently social prescribing interventions supported by GPs, working alongside other healthcare professionals, community groups, and the recipient in the delivery of the intervention, as indicated here: There is also some wider contextual work focusing on the evidence base for the impact of social prescribing on various groups, e.g., the effect on older adults, and community-based older adults with a range of health issues. The final component of the NPT is reflexive monitoring, which includes evaluating and valuing the impact of a new practice as a living complex intervention process (16). The NPT was used to elucidate how perception influences the embedding and effectiveness of arts, nature, and physical activity social prescriptions for psychosocial health and well-being interventions for GPs. Hence, through synthesizing the utility of the four dimensions of coherence, cognitive participation, collective action, and reflexive monitoring in the NPT, healthcare practitioners are positioned to enhance the implementation of social prescription practices to improve the psychosocial health and well-being of patients (see Figure 1).

#### Ethno-psychosocial theory

The ethno-psychosocial concept assumes that an individual has cultural, psychological, and social aspects that shape their actions. Ethnopsychosocial theory is a set of criteria for evaluation that, in general, can assess the level of a general practitioner’s knowledge and ability to make a proper or improper attitude toward the future psychosocial state of a patient. The ‘ethno-psychosocial’ theory assists in gaining awareness about cultural variables and the dyadic relationship between the patient and counselor (general practitioner). It becomes pertinent to discover how culture helps shape people’s perceptual attitudes toward health, beliefs about health, and actions regarding health. Additionally, ethno-psychosocial theory emphasizes a person’s psychological and clinical aspects of health (17). General practitioners understand that evaluating patients’ psychological status can reveal the emotional or cognitive etiology contributing to their concerns. It can help make treatment plans and management procedures that can treat the physical manifestations of the condition. Ethnopsychosocial theory emphasizes social factors as influencing individuals’ health status and related conditions (18). Socioeconomic status, education level, employment status, and other aspects related to access to healthcare services can widely affect one’s health and well-being (19). Furthermore, ethno-psychosocial theory challenges general practitioners in that they listen to their patients and provide them with cloaked information tailored to their needs, desires, and beliefs (20). Through shared decision-making and collaboration with patients in treatment planning, general practitioners positively impact patients’ lives by making them active in the self-management of their health. This makes the treatment relationship an effective collaboration between the GP and the patient so that there will be effective communication, more trust, and increased compliance with treatment. Indeed, patient-centered care also considers patients’ perceptions of their own experience and thus prioritizes their needs and responds accordingly (21). Cultural proficiency involves effectively working with people from different backgrounds and interacting with patients according to their cultural values. There should be a recommendation for the integrated multiprofessional approach in providing psychosocial support with the collaboration of other healthcare providers and associates, social workers, psychologists, and community support organizations. By having colleagues from different disciplines operate in a multifaceted manner, those general practitioners are not only able to draw on the knowledge and outlook of other workers but also able to address all the psychosocial factors affecting their patients effectively and holistically (22). The strengths of interprofessional collaboration include the ability to screen and assess psychosocial aspects of the patient, design integrated care plans, and implement comprehensive services that ensure comprehensive management of the different aspects of the human being. It creates a strong collaborative team setting where general practitioners can turn for support and counseling on general practice and practical information to assist them in providing optimal psychosocial care to their patients. Ethnopsychosocial theory can potentially become a valuable tool for assessing general practitioners’ degree of awareness of their patients’ needs and priorities in terms of their psychosocial context (23) (see Figure 1).

### Statement of the study

**Figure 2:**
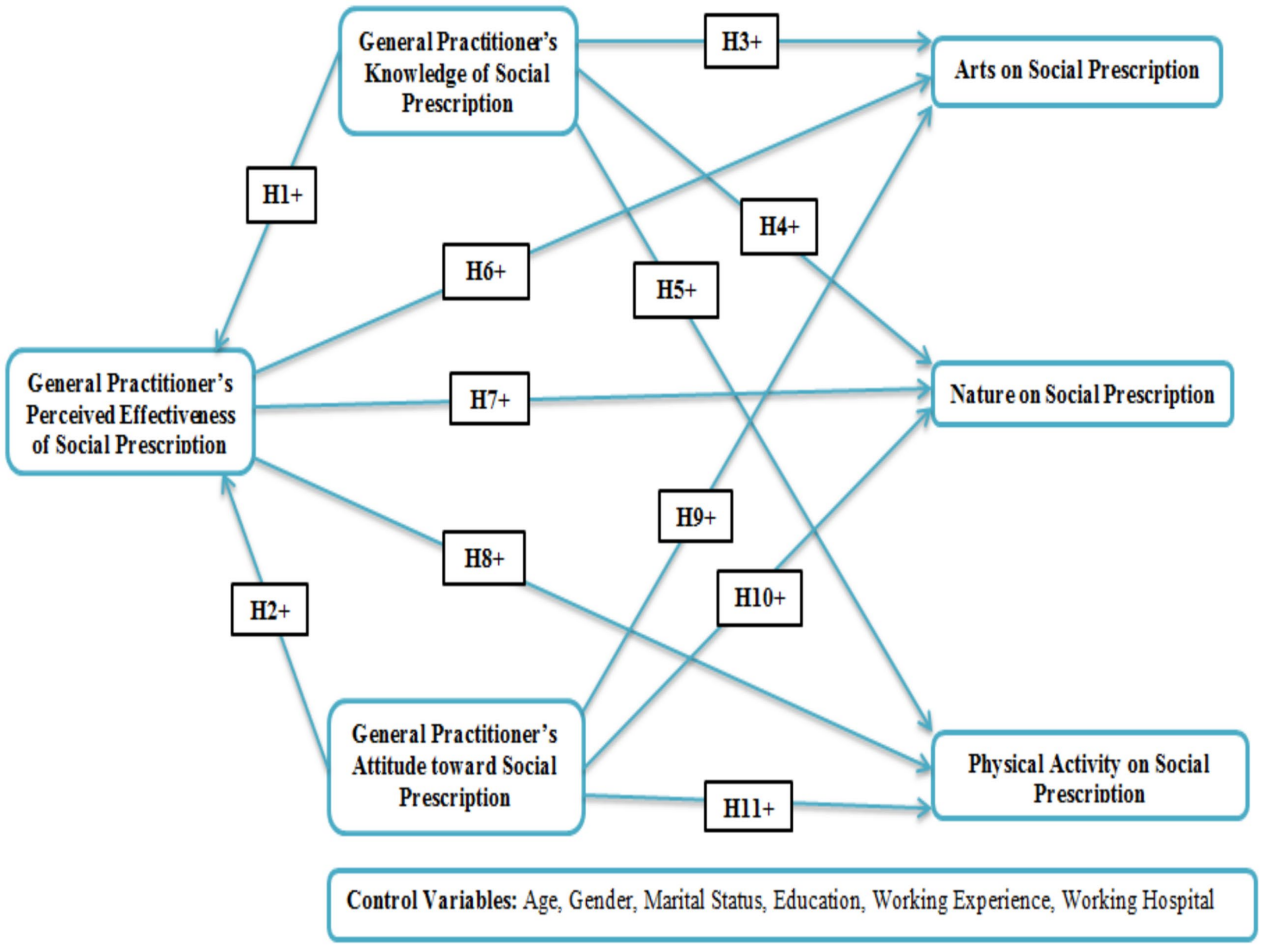
Hypothetical model.

Through social referrals, medical professionals, including general physicians and nurses, can divert patients with social difficulties, such as personal problems, toward nonclinical resources and community-based organizations. General practitioners and primary care services can benefit from this in relieving the extra workload by offering a different and versatile way to address patients’ problems when standard procedures are inappropriate (8). GPs occupy a central position in enforcing the comprehensive well-being of patients in a bid to promote the overall good of patients; social prescription is a new concept that has attracted attention (4). The purpose of a green park’s prescribing practice is to create a list of activities, such as being in touch with arts, nature, and exercise, which benefit mental and physical health (11). Research has shown that participants improve their health and increase social cohesion and community vitality, particularly those who deal with mental health problems (8). Social prescription, focused on well-being, is a holistic concept that involves physical, mental, relational, and emotional aspects in helping people develop and sustain good practices. Therefore, 17 countries have decided to design and apply dedicated social prescribing interventions to address psychological and social factors that could improve health and well-being within the framework of multiple health systems (24). With the help of local community organizations, China has started a community-led social prescribing scheme under a comprehensive evaluation team, which includes general practitioners (25). GPs carry out social prescribing activities effectively because of their close connection to patients. Knowing GPs’ education, thoughts, and viewpoints about their impact on social prescription is the prelude to effectively incorporating this practice into our medical practice. Existing studies (3–5, 7, 9) have highlighted the influence of social, cultural, and environmental circumstances on behavior patterns. Nevertheless, comprehensive research on how these factors impact general practitioners’ attitudes and knowledge in prescribing nonpharmacological interventions is lacking. They primarily work at the primary level (Tier 1) of the healthcare system of China, community health centers (CHCs) and stations within cities, and township health centers (THCs) within rural areas. Their core functions include preventive medicine, initial treatment/triaging of common diseases, management of chronic conditions, health prevention, and making referrals. Therefore, the current study aims to address this research gap by comprehensively understanding the influence of general practitioners’ perceptions (knowledge, attitudes, and perceived effectiveness) on arts, nature, physical activity, and social prescription for psychosocial health and well-being (see Figure 2).

### Hypothesis operationalization

Interest in the role of social prescription as a central part of modern healthcare, particularly in its influence on psychosocial health, is increasing. Healthcare providers’ positive opinions of collaboration help toward a complete collaboration process in which shared decision-making is encouraged in mental health care. This can be explained again by the instrumental cost–benefit reasoning behind the understanding that patients and GPs collaborate to reach better health (26). Educational interventions seem to result in better prescribing between GPs, and such educational input may contribute to the acceptability and effectiveness of social prescribing (27, 28). A previous study indicated that experts with insights into social support dynamics can devise effective social prescriptions for addressing particular clinical requirements of individuals and communities (29). This strongly indicates that encouraging enabling beliefs in social prescriptions drives adherence to such interventions and boosts their psychosocial benefits (30). At the community level, an evidence synthesis with a systematic scoping review demonstrated the relationships among health professionals’ feelings about social prescribing and patient outcomes, reduced loneliness, and enhanced community integration of vulnerable populations (31). The evidence suggests that a GP’s knowledge about social prescribing could influence the perceived effectiveness of SP for psychosocial health and well-being. The following hypotheses were proposed on the basis of the literature to achieve the study objective.

*H*1: A positive association exists between general practitioner knowledge regarding social prescription and the perceived effectiveness of social prescription for psychosocial health and well-being.

*H*2: A positive association exists between general practitioners' attitudes regarding social prescription and the perceived effectiveness of social prescription in psychosocial health and well-being.

Art-based interventions in social prescriptions have made a significant difference in individuals’ mental and psychosocial health. A study by Jensen et al., indicated that arts-on-prescription could positively impact an essential improvement in mental health, specifically emotional well-being (32). Furthermore, GPs who are informed about social prescriptions are well placed to recommend creative interventions that encourage people to ‘feel’ their anxiety and depression as part of emotional regulation (8). Arts-based activities can play a therapeutic role and promote socialization, and they are integral in overcoming isolation and fostering a sense of community (8). Nature-based activities have been successful in people who are lonely and socially isolated, especially older people (31). GPs who are knowledgeable about nature-based interventions can advocate for prescribing, which may result in more positive patient outcomes (33, 34). These findings are supported by the literature, which confirms that GPs willing to prescribe PA are more likely to promote the health behaviors required for mental health promotion (35). It is also responsible for GPs to nurture an understanding of social prescription practices and how their understanding influences arts, nature, and exercise for psychosocial health and well-being. The following hypotheses were proposed on the basis of the literature to achieve the study objective.

*H*3: Social prescription knowledge by the general practitioner has a positive effect on the arts on social prescription for psychosocial health and well-being.

*H*4: General practitioners' knowledge regarding social prescription is positively associated with the nature of social prescription in psychosocial health and well-being.

*H*5: Knowledge of the social prescription of a general practitioner has a positive effect on physical activity on social prescription for psychosocial health and well-being.

Social prescription is led by general practitioners (GPs), and it is assumed that perceived effectiveness will determine the implementation and embedding of working practices. Engagement with the arts, including arts interventions, has been identified as significantly impacting mental health and well-being. Participatory arts-based interventions, for example, have improved the resilience and perceptions of social connectedness among participants, particularly those in vulnerable subgroups (36). Arts on prescription have been shown to be powerful modalities that significantly improve one’s health and well-being. Consequently, many GPs endorse arts-based prescriptions in medical settings (32). The narrative of the social prescription context, especially of arts, nature, and physical activity, is that GPs can engage as they perceive that, through the domain, perceived improvement can be achieved. A change in perceived effectiveness can occur. Incorporating these factors into treatment paradigms benefits patient health outcomes and may help GPs feel that they are prescribing more effectively. Greater knowledge of local resources and how they can be incorporated into patient care might lead to increased use of social prescribing (37). On the basis of the extent of available evidence from the literature, they have different implications for mental and physical health and perceived efficacy by health workers. The following hypotheses were proposed on the basis of the literature to achieve the study objective.

*H*6: The perceived effectiveness of social prescription for psychosocial health and well-being by the general practitioner and arts on social prescription has a positive association.

*H*7: Perceived effectiveness of social prescription by the general practitioner for psychosocial health and well-being, and the nature on social prescription has a positive association.

*H*8: Perceived effectiveness of social prescription by the general practitioner for psychosocial health and well-being, and physical activity in social prescription has a positive association.

Social prescribing, where GPs can prescribe to patients a nonclinical service/activity for psychosocial benefit, has the potential to be an innovative intervention. The beliefs of GPs toward the arts and related activities are likely to be a significant determinant of the capacity for social prescribing to impact patients’ psychosocial well-being. Engaging in the arts is known to have a beneficial effect on mental health. For example, integrated arts-based interventions in medical wards have been reported to have beneficial effects on symptoms of anxiety and depression, resulting in improved psychosocial outcomes (37). When GPs facilitate art-based prescriptions, the likelihood that patients engage in such interventions increases, benefiting patient well-being (34). The opinion of GPs regarding social prescriptions is a significant determinant of whether they are likely to be used and, thus, whether referrals will filter through and influence patient outcomes. A supportive stance by GPs with a special interest in nonmedical options establishes a new balanced patient–doctor relationship and is a prerequisite for successful social prescribing (34). GP active champions for these interventions might promote patient adherence and provide a way for models of care where mental health is considered as important as physical health (38). The extent to which arts and physical activity are conceptualized by GPs can significantly impact the precision and potential benefit of social prescriptions regarding psychosocial health and well-being. Through more optimism and the setting of limits in the interventions, the GP, in this manner, supports better health with a higher probability of a good quality of life for the patient. The following hypotheses were proposed on the basis of the literature to achieve the study objective.

*H*9: The attitudes of the general practitioner are positively associated with arts on social prescription for psychosocial health and well-being.

*H*10: The attitudes of the general practitioner are positively associated with the nature of social prescription for psychosocial health and well-being.

*H*11: The attitudes of the general practitioner are positively associated with physical activity related to social prescription for psychosocial health and well-being.

## Methods

The study was conducted in China. The Fourth Affiliated Hospital of Zhejiang University School of Medicine in Yiwu, Zhejiang, China, was granted ethical approval (K2023034). In addition, the study also upheld the Helsinki Declaration.

### Study design and participants

The research was exploratory, and a cross-sectional survey design was adopted, aiming to collect data at a single point in time from a sample of general practitioners in China. The study participants gave their informed consent after fulfilling the eligibility requirements (participants must be 25 + years old, have a minimum of 3–5 years of general practice experience, possess knowledge of social prescription, and work in public settings involved in the social prescription for health and well-being). A total of 701 general practitioners were recruited via a simple random stratified sampling technique that fulfilled the inclusion criteria (Table 1).

**Table 1:** Demographics (N-701).

Variables	Categories	Frequency/Percentage
Gender	Male	363 (51.78%)
	Female	338 (48.22%)
Marital status	Single	254 (36.23%)
	Married	343 (48.93%)
	Divorced	82 (11.7%)
	Widowed	22 (3.14%)
Educational qualification	Bachelor	281 (40.09%)
	Master	280 (39.94%)
	PhD	140 (19.97%)
	Other	0 (0.0%)
Geographic location (China)	Northern	95 (13.55%)
	Eastern	91 (12.98%)
	Central	83 (11.84%)
	Southern	73 (10.41%)
	Northeast	87 (12.41%)
	Northwest	75 (10.7%)
	Southeast	197 (28.1%)
Working experience	3–5 years	150 (21.4%)
	6–10 years	279 (39.8%)
	11–15 years	151 (21.54%)
	16 Years+	121 (17.26%)
Working hospital	Tertiary level	248 (35.38%)
	Secondary level	213 (30.39%)
	Primary level	240 (34.24%)

### Data collection tool construction and validation

The primary data were gathered via a self-administered questionnaire survey. It was constructed via a five-step sequential methodology, ensuring the reliability and validity of the instrument (39). After the items were created, a test group comprising 25 people evaluated the questionnaire. Pilot testing can help identify any problems related to the language used in the questions, the answer possibilities, or the clarity of the questions (40). In response to feedback from the pilot study, we revised the questionnaire to increase its user-friendliness. The final questionnaire survey comprised 35 items, each assigned a Likert scale score.

### Survey measures

#### Demographics

The demographic section contains questions related to gender, marital status, education, region, working experience, and working hospital.

#### General practitioners’ knowledge of social prescription

Social prescription is a technique that has gained popularity for treating nonmedical reasons for illness. It connects primary care patients with community and volunteer resources to enhance their health, well-being, and care experience (41). Most consultations with GPs are for psychosocial rather than medical issues, which is noteworthy (42). This makes social prescribing a crucial tool for helping medical professionals address the nonmedical causes of illness with nonmedical interventions (8). Social prescribing has been explored in the context of public health initiatives embedded within general practice, aligning with local and national agendas to improve health and well-being and reduce health inequalities (43). GPs must comprehend social prescription well to use it efficiently in their clinical setting (3, 4, 9, 12, 44). The GPs’ knowledge of social prescription for psychosocial health and well-being was assessed via a five-point Likert scale (see the Supplementary material).

#### General practitioners’ attitudes toward social prescription

Psychosocial health and well-being are linked with social determinants of health. Social prescribing is a holistic approach to addressing the social determinants of health to improve psychosocial health quality. The GP’s attitude toward social prescription implementation impacts recommended activities and patient outcomes (45). They also involve themselves in holistic care and are critical players among patients. GPs are positioned at the forefront of guiding the social prescribing process, and positive attitudes are the key to their success. Research has shown that general practitioners’ attitudes and knowledge contribute to the vital role played by the policies and practices promoting health and well-being (46). The druggists’ attitudes toward pharmacotherapy, which are observed to have substantial control over prescription practices, indicate that individual physicians’ attitudes play a considerable role in medical decision-making (45–47). The GPs’ attitudes toward social prescription for psychosocial health and well-being were assessed via a five-point Likert scale (see the Supplementary File).

#### General practitioners’ perceived effectiveness of social prescription

Healthcare providers work with linked workers by developing nonclinical social prescriptions, through which it is possible to refer patients to social prescriptions for their well-being and overall health (48). Social prescribing, which has been connected with community support for individuals and the general population, is one way to achieve increased health and well-being (49). General practitioners evaluate social prescription as a pragmatic method emerging as a primary tool and a means of general improvement for patients who run parallel with healthcare. Social prescription allows GPs to address medical needs and social, psychological, and lifestyle factors influencing health. After pertinent studies were reviewed (3, 4, 48, 49), a five-point Likert scale was used to assess GPs’ perceived effectiveness of social prescription for psychosocial health and well-being (see the Supplementary File).

#### Arts on social prescription

The report titled “Arts for Health and Well-Being” highlights the positive effects of the arts on one’s physical, psychological, and social development (50). GPs recognize the therapeutic benefits of engaging in artistic activities, such as painting, music, dance, and theatre, in improving mental health, reducing stress, and enhancing overall quality of life. They perceive the arts on social prescription as a holistic approach that complements medical treatments by addressing social and emotional needs. A five-point Likert scale was developed after reviewing the pertinent studies (8, 50–52) to assess the GP’s opinions about the arts on social prescription for psychosocial health and well-being (see the Supplementary File).

#### Nature on social prescription

Nature-based interventions have gained attention as potential approaches for improving health and well-being, particularly mental health. There is growing interest in the potential for outdoor interventions in natural settings to improve health and well-being, particularly in treating and preventing depression (53). Interventions rooted in nature have been linked to gains in mental, bodily, and social well-being (54). Contact with nature has been shown to positively affect people’s health by reducing stress (55). GPs understand the profound benefits of connecting with nature for physical and mental health. They view gardening, hiking, forest bathing, and other nature-based activities as powerful ways of reducing stress and enhancing quality of life. GPs have supported nature in terms of social prescription because they provide a holistic model in healthcare and consider social, psychological, and environmental aspects. The term “green prescriptions” arises from a written prescription on lifestyle changes, particularly physical activity, provided by health practitioners (56). A five-point Likert scale was developed after reviewing the pertinent studies (8, 53–56) to assess the GPs’ opinions regarding the nature on social prescription for psychosocial health and well-being (see the Supplementary File).

#### Physical activity on social prescription

Social prescribing has been recognized to include the prescription of physical activities for health benefits. GPs have been found to prescribe physical activity and exercise through the “Green Prescription” program to promote physical activity and improve health (57). The prescription of physical activities by general practitioners has been a concern in promoting health and well-being. Integrating physical activity within social prescriptions offers a promising approach through which general practitioners can promote health and well-being (58). The GPs’ opinions regarding physical activity on social prescription for psychosocial health and well-being were rated via a five-point Likert scale developed after the relevant pertinent studies were reviewed (see the Supplementary File).

### Data collection and analysis

An online survey was conducted to collect the primary data. The online poll followed the CHERRIES guidelines (59). The data analysis for this inquiry used the Smart-PLS 3.2.9 software package (60). The data analysis approach used a biphasic methodology. First, the construct, reliability, and convergent validity were assessed via a measuring technique (see Table 2). A structural model was developed in the second stage to empirically analyze the proposed theories (61, 62).

**Table 2:** Validity and reliability tests.

Items	Mean	Standard deviation	Excess Kurtosis	Skewness
GPK1	3.611	1.279	−0.765	−0.559
GPK2	3.368	1.179	−0.704	−0.263
GPK3	3.635	1.288	−0.877	−0.525
GPK4	4.031	1.119	0.043	−0.962
GPK5	3.428	1.271	−0.969	−0.344
GPA1	3.573	1.279	−0.731	−0.578
GPA2	3.354	1.169	−0.616	−0.254
GPA3	3.563	1.281	−0.836	−0.482
GPA4	3.961	1.151	−0.42	−0.814
GPA5	3.412	1.296	−0.994	−0.326
GPPESP1	3.623	1.292	−0.667	−0.629
GPPESP2	3.407	1.122	−0.562	−0.29
GPPESP3	3.666	1.248	−0.797	−0.536
GPPESP4	4.039	1.109	−0.002	−0.963
GPPESP5	3.441	1.273	−0.903	−0.374
GPPESP6	3.578	1.268	−0.706	−0.554
GPPESP7	3.387	1.136	−0.52	−0.336
GPPESP8	3.573	1.305	−0.91	−0.48
GPPESP9	4.014	1.111	−0.198	−0.884
GPPESP10	3.409	1.286	−0.928	−0.361
NoSP1	3.971	1.155	−0.058	−0.918
NoSP2	3.625	1.278	−0.853	−0.51
NoSP3	3.578	1.277	−0.871	−0.466
NoSP4	4.003	1.142	−0.081	−0.927
NoSP5	3.923	1.199	−0.15	−0.906
AoSP1	3.612	1.253	−0.698	−0.576
AoSP2	3.392	1.144	−0.606	−0.31
AoSP3	3.552	1.333	−0.943	−0.474
AoSP4	4.004	1.095	−0.111	−0.89
AoSP5	3.431	1.227	−0.827	−0.322
PAoSP1	3.566	1.308	−0.907	−0.486
PAoSP2	3.308	1.139	−0.638	−0.171
PAoSP3	3.546	1.276	−0.927	−0.393
PAoSP4	3.971	1.12	−0.292	−0.817
PAoSP5	3.417	1.235	−0.869	−0.334

GPK, general practitioner knowledge; GPA, general practitioner attitude; GPPESP, general practitioner perceived effectiveness of social prescription; AoSP, arts on social prescription; NoSP, nature on social prescription; PAoSP, physical activity on social prescription.

## Results

### Validity and reliability tests

Table 2 provides a complete summary of the statistical information for several items or variables, including their relative means, standard deviations, excess kurtosis, and skewness. The statistics below enhance the understanding of each item’s characteristics and data distribution.

### Factor analysis

**Figure 3:**
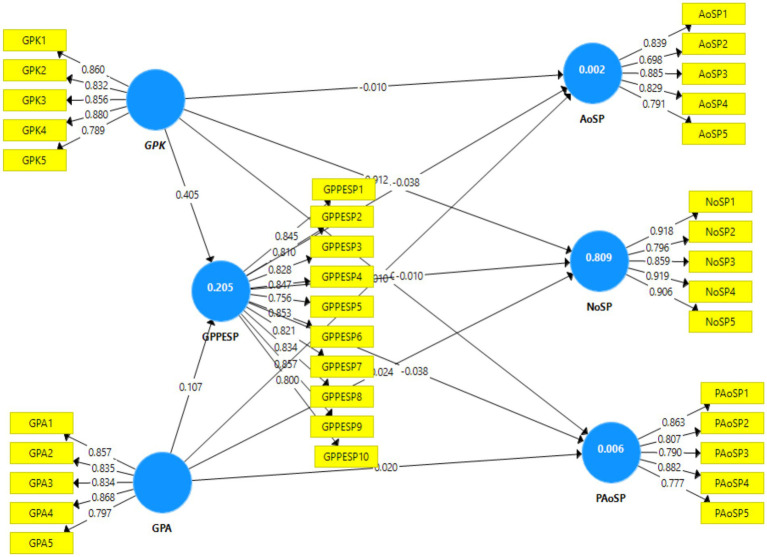
Model measurement.

The measurement model must fulfill the criteria for convergent and discriminant validity. Convergent validity is established when the loading is equal to or greater than 0.70, the average variance extracted (AVE) is 0.5, and the composite reliability (CR) achieves a minimum value of 0.7 (63). Table 3 and Figure 3 confirm the acceptable convergent validity of the research by demonstrating that the variance inflation factor (VIF), AVE and CR are more significant than the threshold values are. This indicates that there are no issues with the convergent validity of the study’s construct.

**Table 3:** Factor analysis (N701).

Constructs	Items	Loadings	VIF	Alpha	CR	AVE
General practitioner’s knowledge of social	GPK1	0.860	3.306	0.899	0.925	0.712
	GPK2	0.832	4.166			
	GPK3	0.856	7.839			
	GPK4	0.880	6.169			
	GPK5	0.789	3.326			
General practitioner’s attitude to social prescription	GPA1	0.857	3.783	0.894	0.922	0.703
	GPA2	0.835	2.967			
	GPA3	0.834	3.201			
	GPA4	0.868	3.733			
	GPA5	0.797	3.644			
General practitioners’ perceived effectiveness of social prescription	GPPESP1	0.845	3.918	0.948	0.949	0.682
	GPPESP2	0.810	4.366			
	GPPESP3	0.828	4.374			
	GPPESP4	0.847	5.041			
	GPPESP5	0.746	2.783			
	GPPESP6	0.853	5.535			
	GPPESP7	0.821	3.670			
	GPPESP8	0.834	4.303			
	GPPESP9	0.857	4.468			
	GPPESP10	0.800	2.748			
Arts on social prescription	AoSP1	0.839	2.633	0.885	0.905	0.658
	AoSP2	0.698	3.280			
	AoSP3	0.885	7.318			
	AoSP4	0.829	2.347			
	AoSP5	0.791	2.638			
Nature on social prescription	NoSP1	0.918	3.440	0.927	0.945	0.776
	NoSP2	0.796	3.686			
	NoSP3	0.859	3.471			
	NoSP4	0.919	3.902			
	NoSP5	0.906	2.811			
Physical activity on social prescription	PAoSP1	0.863	2.573	0.884	0.905	0.680
	PAoSP2	0.807	3.231			
	PAoSP3	0.790	3.712			
	PAoSP4	0.882	3.584			
	PAoSP5	0.777	3.254			

### Discriminant validity and convergent validity

Discriminant validity (DV) was used to examine and define distinct notions. The DV also confirms all the measurements of the component differences. The discriminant validity of a factor was calculated via its average variance extracted (AVE). The discriminant validity results revealed that the square root of each construct and average variance extracted (AVE) had higher values than their correlations with other constructs (see Table 3). In structural equation modeling (SEM), the Heterotrait–Monotrait ratio of correlations (HTMT) measures discriminant validity across constructs (64). The HTMT ratio compares the correlations of items assessing two constructs. The HTMT ratios in the table indicate that the constructs under study have valid discriminant validity (see Table 4). Model fitness was assessed by calculating SRMR, d_ULS, d_G, chi-square, and NFI. The model fitness statistics show that the proposed model of the study is acceptable for structural equation modeling (see Table 4).

**Table 4:** Discriminant and convergent validity and model fit summary (N701).

Fornell–Larcker criterion						
Constructs	AoSP	GPA	GPK	GPPESP	NoSP	PAoSP
AoSP	0.811					
GPA	−0.003	0.839				
GPK	−0.024	0.342	0.844			
GPPESP	−0.04	0.246	0.441	0.826		
NoSP	−0.003	0.286	0.899	0.387	0.881	
PAoSP	0.311	−0.01	−0.07	−0.06	−0.046	0.825

HTMT						
Constructs	AoSP	GPA	GPK	GPPESP	NoSP	PAoSP
AoSP	—					
GPA	0.035					
GPK	0.036	0.382				
GPPESP	0.038	0.267	0.476			
NoSP	0.032	0.312	0.971	0.41		
PAoSP	0.34	0.037	0.08	0.062	0.054	—

Model fit summary	
Statistical tests	Estimated model
SRMR	0.045
d_ULS	0.809
d_G	0.167
Chi-Square	4655.08
NFI	0.899

GPK, general practitioner knowledge; GPA, general practitioner attitude; GPPESP, general practitioner perceived effectiveness of social prescription; AoSP, arts on social prescription; NoSP, nature on social prescription; PAoSP, physical activity on social prescription; SRMR, standardized root mean square residual; d_ULS, squared Euclidean distance; d_G, geodesic distance; NFI, normed fit index.

### Hypothesis testing

**Figure 4:**
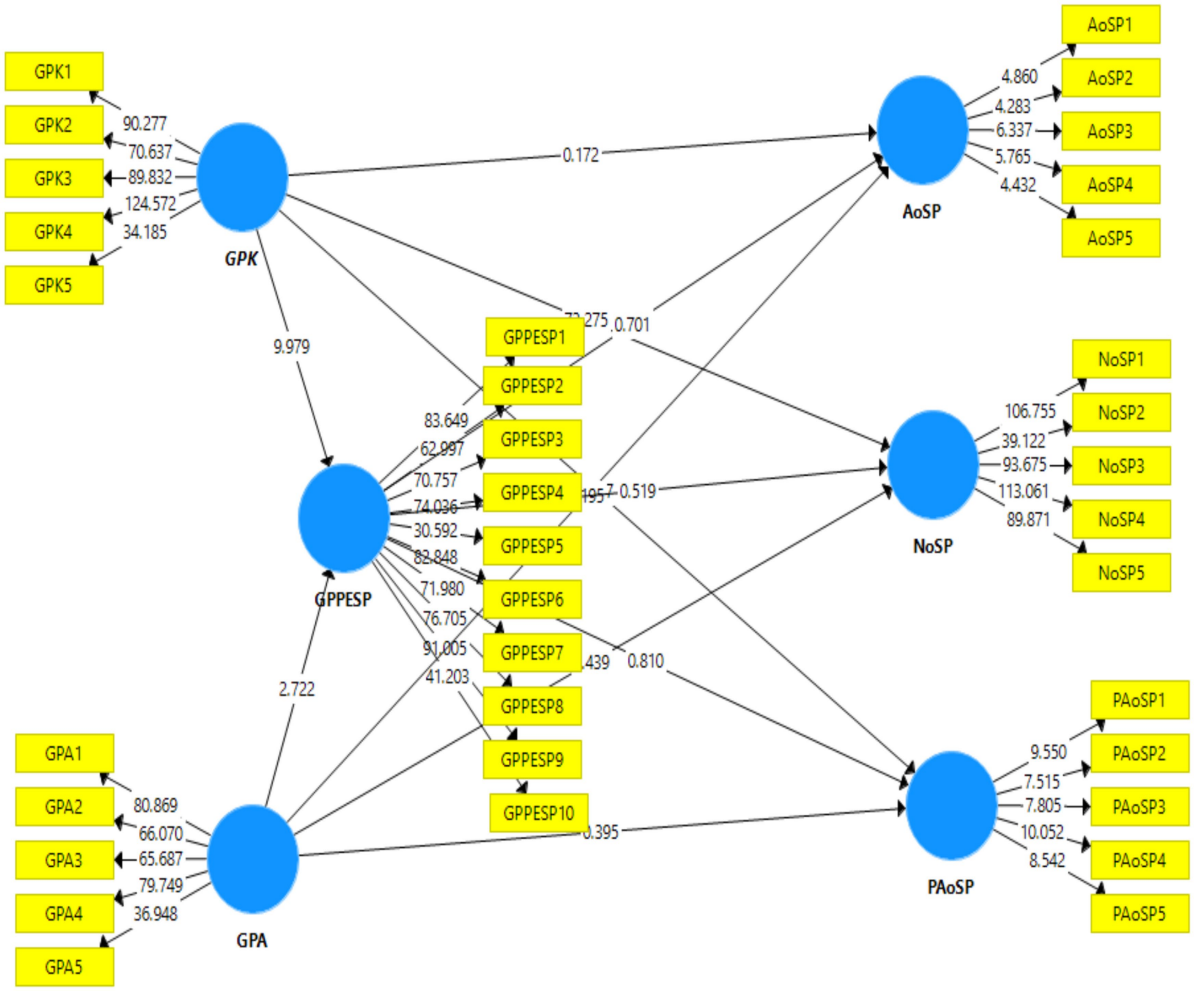
Path analysis.

All hypotheses demonstrated statistically significant relationships, as indicated by *p*-values of < 0.001 (see Table 5 and Figure 4).

**Table 5:** Hypothesis testing results.

Hypotheses	Sample mean (M)	Standard deviation (STDEV)	T statistics (|O/STDEV|)	Beta values	*P* values	Decision
H1: GPK → GPPESP	0.402	0.039	10.418	0.405	0.001	Confirm
H2: GPA → GPPESP	0.108	0.039	2.772	0.107	0.001	Confirm
H3: GPK → AoSP	−0.008	0.061	0.172	−0.010	0.001	Confirm
H4: GPK → NoSP	0.913	0.012	79.05	0.002	0.001	Confirm
H5: GPK → PAoSP	−0.063	0.047	1.276	0.049	0.001	Confirm
H6: GPPESP → AoSP	−0.033	0.058	0.655	0.000	0.001	Confirm
H7: GPPESP → NoSP	−0.011	0.018	0.540	1.024	0.001	Confirm
H8: GPPESP → PAoSP	−0.04	0.05	0.767	0.120	0.001	Confirm
H9: GPA → AoSP	0.01	0.048	0.201	0.000	0.001	Confirm
H10: GPA → NoSP	−0.024	0.017	1.409	0.110	0.001	Confirm
H11: GPA → PAoSP	0.024	0.051	0.395	0.013	0.001	Confirm

GPK, general practitioner knowledge; GPA, general practitioner attitude; GPPESP, general practitioner perceived effectiveness of social prescription; AoSP, arts on social prescription; NoSP, nature on social prescription; PAoSP, physical activity on social prescription.

*Hypothesis* 1 (GPK → GPPESP): A positive association exists between general practitioner knowledge regarding social prescription and the perceived effectiveness of social prescription for psychosocial health and well-being

The findings of H1 (*t* = 10.418, *β* = 0.405, *p* < 0.001) indicate a positive relationship, implying that higher levels of knowledge among general practitioners are associated with greater perceptions of the effectiveness of social prescriptions in enhancing psychosocial outcomes.

*Hypothesis* 2 (GPA → GPPESP): A positive association exists between general practitioners' attitudes regarding social prescription and the perceived effectiveness of social prescription in psychosocial health and well-being.

The findings of H2 (*t* = 2.772, *β* = 0.107, *p* < 0.001) indicate that more favorable attitudes toward social prescriptions correlate with heightened perceptions of their effectiveness in psychosocial well-being.

*Hypothesis* 3 (GPK → AoSP): The general practitioner’s social prescription knowledge has a positive effect on the arts’ social prescription for psychosocial health and well-being.

The findings for H3 (*t* = 0.172, *β* = −0.010, *p* < 0.001) suggest a very small inverse relationship. This implies that greater knowledge may slightly diminish the emphasis on arts-based social prescriptions, possibly because practitioners prioritize other modalities or perceive art as less central to psychosocial benefits.

*Hypothesis* 4 (GPK → NoSP): General practitioners' knowledge regarding social prescription is positively associated with the nature of social prescription in psychosocial health and well-being.

H4 (*t* = 79.05, *β* = 0.002, *p* < 0.001) suggests that knowledge is positively linked to nature-based social prescriptions. This means that knowledge enhancement directly influences the promotion of nature-oriented interventions for psychosocial health.

*Hypothesis* 5 (GPK → PAoSP): Knowledge of the social prescription of a general practitioner has a positive effect on physical activity on social prescription for psychosocial health and well-being.

The findings of H5 (*t* = 1.276, *β* = 0.049, *p* < 0.001) indicated that increased knowledge enhances physical activity as a social prescription. This finding implies that knowledge-building efforts encourage practitioners to integrate physical activity to improve psychosocial well-being.

*Hypothesis* 6 (GPPESP → AoSP): The perceived effectiveness of social prescription for psychosocial health and well-being by the general practitioner and arts on social prescription has a positive association.

The findings of H6 (*t* = 0.655, β = 0.000, p < 0.001) indicate a statistically significant but minimal effect of the perceived effectiveness of social prescription on psychosocial health and well-being by the general practitioner and arts on social prescription.

*Hypothesis* 7 (GPPESP → NoSP): Perceived effectiveness of social prescription by the general practitioner for psychosocial health and well-being, and the nature on social prescription has a positive association.

The findings of H7 (*t* = 0.540, *β* = 1.024, *p* < 0.001) point a strong positive association. This implies that a higher perceived effectiveness substantially bolsters nature-based social prescriptions. This relationship highlights the potential of efficacy-focused interventions to amplify the use of nature-oriented approaches in psychosocial care.

*Hypothesis* 8 (GPPESP → PAoSP): Perceived effectiveness of social prescription by the general practitioner for psychosocial health and well-being, and physical activity in social prescription has a positive association.

The findings for H8 (*t* = 0.767, *β* = 0.120, *p* < 0.001) indicate a small positive effect. This means that stronger perceptions of effectiveness are linked to greater integration of physical activity as a social prescription for psychosocial benefits.

*Hypothesis* 9 (GPA → AoSP): The attitudes of the general practitioner are positively associated with arts on social prescription for psychosocial health and well-being.

Findings for H9 (*t* = 0.201, *β* = 0.000, *p* < 0.001). This finding implies the need to explore other factors that drive the adoption of art interventions in psychosocial health contexts.

*Hypothesis* 10 (GPA → NoSP): The attitudes of the general practitioner are positively associated with the nature of social prescription for psychosocial health and well-being.

The findings for H10 (*t* = 1.409, *β* = 0.110, *p* < 0.001) reflect a small positive association. This suggests that positive attitudes modestly enhance nature-based social prescriptions, pointing to attitude modification as a strategy to increase the utilization of psychosocial well-being.

*Hypothesis* 11 (GPA → PAoSP): The attitudes of the general practitioner are positively associated with physical activity related to social prescription for psychosocial health and well-being.

The findings of H11 (*t* = 0.395, *β* = 0.013, *p* < 0.001) showed a significant relationship between general practitioner attitudes and physical activity as social prescriptions. This implies that attitudes have a direct impact on physical activity as social prescriptions.

These findings highlight the multifaceted roles of knowledge, attitudes, and perceived effectiveness in shaping social prescription practices. Overall, the results affirm the theoretical model, with GPK and GPSE generally exhibiting stronger effects than GPA.

## Discussion

Social prescribing is a nonpharmacological intervention that has recently been introduced in primary care. In the integrated model developed, the general practitioner is incorporated into the allied health staff, maintaining a strong link with referrals to community-based programmes. Evidence of the programmes has been based on reducing the psychosocial risks of loneliness, anxiety, depression, and low life satisfaction. Social prescribing is most often used to refer patients to arts projects, outdoor activities, and formalized exercise (65). These interventions incorporate both mental and physical health, which is frequently overlooked in other interventions. They also build social cohesion and deliver holistic care. Social prescribing depends on the significance of GP attitudes and perceptions. However, the different perceptions of the GPs regarding their professional competence, experience, and the perceived effectiveness of their role affect referral patterns and, therefore, have a direct influence on programme effectiveness. It is thus essential that, to implement social prescribing effectively and deliver the maximum benefits to referrers, clinicians understand how a GP views social prescribing and how to introduce it as part of daily primary care practice (65).

In primary care, social prescribing involves health professionals, particularly GPs, who refer patients to nondrug interventions to increase their health and well-being (66). Social prescribing is a nonclinical approach to address psychosocial aspects of well-being, reduce the workload of GPs, and promote well-being by directing patients to community services (67). GPs consider the physical symptoms and social, emotional, and psychological factors that impact their patients’ health and well-being. Once patients’ needs are identified, GPs can connect them with appropriate social prescribing services and resources within the community (68). Our study results show that GPs’ knowledge is vital in implementing social prescriptions for patients’ health and well-being. GPs play a crucial role in social prescribing by integrating social and environmental factors into their decisions (7). GPs implement social prescribing services in general practice settings and evaluate their effects on patient well-being and primary care resource use (68). Social prescribing interventions improve patients’ well-being outcomes for those with mild mental health issues and isolation by giving them a support system and empowering them to think about other options for managing their health (69).

Our findings reveal that healthcare providers’ expert knowledge and attitudes contribute significantly to social prescription implementations and their choices during various social prescriptions, such as arts, nature, and physical activity, for psychosocial health and well-being. GPs’ ability to consider social prescription procedures takes place from their position as the ones they can rely on the most within the healthcare system. GPs now recommend creativity, including participation in the arts as performers or audience, to prevent illnesses and help patients’ general wellness (69). General practitioners are the primary care health providers who serve on the front line and have an extraordinary blend of knowledge, attitudes, and social skills designed for application in social prescription practices (70, 71).

Studies have shown that activities designed to be artistic, such as painting, drawing, and sculpting, can heal people with psychological issues (52, 72). Through arts on social prescription, one of the main benefits is that the arts can help individuals deal with difficulties such as anxiety, depression, and stress. When people engage in different pursuits, such as community theatre, choir, or cooperative art projects, they establish a feeling of belonging and connectedness with their fellow participants at the end of their lives. Our study results reveal the perceived effectiveness of various types of social prescriptions by GPs and the sense of control in determining what practitioners do or refrain from in treatment. Ownership is among the vital aspects that define a particular community’s direction of social medicine. Another key feature is that community-based arts therapies encourage social engagement and community involvement, which are essential for reducing loneliness and isolation. All kinds of outdoor art activities, such as nature photography and botanical sketching, contribute to one’s physical fitness, and people can enjoy their environment closely, making them fall in love with nature (52).

There have been significant developments in integrating arts into social prescription programs offered by general practitioners, which have put them on the map as nonclinical community interventions with different health and wellness needs as a solution (50). Our findings show that GPs’ knowledge and attitudes play a vital role in determining the applicability of the arts on social prescription for patients. The idea is limited in its applicability, as primary care physicians are only empowered to insert art-related activities such as dances, films, music, and painting into people’s lives to address mental problems, social isolation, and other health needs. The perceived success of social prescription, seen in successful patient outcomes, cements the confidence of the GPs in recommending such interventions, thus cultivating a culture in which the arts are a valuable tool in health and well-being enhancement through social prescription. Research has revealed that arts-based interventions involving prescriptions did better than regular interventions did, especially for older adults, whose psychological well-being significantly improved (73). Art on prescriptions varies and includes various activities focused on health, education, and skill development (74). In a cross-sectional survey (*n* = 208 general practitioners from 33 European Countries on social prescribing), 56% of the participants stated that they knew the term social prescribing. A wide disparity was found between countries, with the United Kingdom, Belgium, and Ireland having done the best, while other countries lagged. From a knowledge distribution perspective, seven out of 10 rural practitioners were found to be significantly more aware (75% awareness) than the same group of practitioners in urban (56%) and semi-rural (43%) areas, prompting the need for a contingent geographical model for knowledge distribution. These findings show an overall positive attitude towards social prescribing as a profession-based intervention among GPs. Results show that clinicians perceive this practice as a good psychosocial management. Due to questions from the same questionnaire, 84% of referring GPs indicated that social prescribing can improve the health and well-being of their patients, and 68% felt they had experienced an increase in job satisfaction. GPs believe that the social prescribing model can contribute to addressing psychosocial issues as it includes community engagement, eliminates the pharmaceutical management of the condition, and supports the self-efficacy of the patient (75).

GPs are increasingly prescribing nature-based interventions as part of social prescriptions to improve their patients’ physical and mental health. The prescriptions include gardening, walks in parks, outdoor mindfulness sessions, or conservation projects. Our findings imply that a positive view of social prescription and an understanding of its success further motivate GPs toward including arts-based activities in their treatment programs. Physical activity and exercise prescriptions are acceptable and feasible for general practitioners and patients (76). Walking or cycling in green spaces promotes cardiovascular health and encourages individuals to live more actively. Unlike people who stopped moving together, participants in the trial exhibited moderate increases in physical activity over the short term, with a “generic prescription of exercise” being enhanced by printed materials (77). Our findings suggest that knowledge, attitudes, and perceived effectiveness directly shape the nature of such interventions. The concept of nonpharmaceutical intervention involving activity prescription by general practitioners has revealed the possibility of achieving physical activity among patients, especially passive patients. The results showed that healthcare professionals’ high confidence in how social prescriptions can promote both general health and well-being is an excellent way to increase the trust and motivation levels of their patients and, therefore, encourage more patients to participate in physical activity programs, which are recommended in this way. The healings of nature are recognized quickly, with many opportunities for relaxation, physical activities, and social interaction (10, 56, 78).

## Conclusion

The role of GPs is fundamental for the success of social prescribing strategies, and their involvement ultimately improves healthcare system efficiency. GPs take a more critical position when combined with the prescription of social activities in clinical settings for the social determinants of health. Recognizing attitudes, practitioners’ perceived swaps, barriers to their rights, and the effectiveness of the practices utilized in their decision-making are significant in modifying their prescription habits. The professionals’ knowledge, attitudes, and practices related to social prescription are complex, multiple, and complex. GPs with complete information about options presented by these procedures and a great interest in their efficiency will be inclined to promote these interventions to their patients. GPs, with the knowledge, attitudes, and perceived efficacy of arts, nature, and physical activity, can prescribe these activities to enhance psychosocial health and well-being and significantly impact the prescription’s success.

### Limitations

This study is an essential contribution to the existing literature on social prescription from the perspective of primary care physicians, but it has methodological limitations. Given that the investigation was cross-sectional, it is only possible to conclude an association at one point in time, without any possibility of inferring causality. Consequently, the temporal dynamics cannot be determined, and the danger of reverse causation cannot be excluded. Reliance on self-reported questionnaire data brings the potential for bias due to response bias, specifically the social desirability effect and recall bias, which can affect the fidelity of measurements directed toward the knowledge, attitude, and perceived efficacy. The analytic sample included 701 general practitioners working in the public sector of China who were recruited according to pre-specified inclusion criteria (aged 25 years or older, had 3–5 years of clinical experience, and had previous experience of social prescription). Accordingly, the external validity of the findings is potentially limited when applying them to other settings, such as the private sector, different urban and rural contexts, and foreign jurisdictions, where cultural, systemic, and policy variables may vary and, in turn, impact physicians’ perceptions. The use of an online CHERRIES-based survey runs the risk of selection bias in favor of those respondents with enhanced technological competencies. Moreover, although the structural equation model has been empirically validated and found reliable, adjustments for numerous potential confounders, such as demographic and extrinsic factors known to influence prescribing behavior, were absent. This omission may cover up subtler nuances in the relations. To overcome these limitations, future studies will be longer, employ mixed-methods research designs, and recruit more participants from diverse ethnic backgrounds. Implementing more rigorous and comparable techniques will increase the robustness and generalizability of the study’s findings and participants’ responses.

## Data Availability

The raw data supporting the conclusions of this article will be made available upon a reasonable request to the corresponding authors.
