# Supplementary material for: Influence of general practitioners’ perceptions on social prescription of arts, nature, and physical activity for psychosocial health and well-being: a structural equation model approach
Source: Front Public Health. 2025 Dec 10;13:1649931. doi: 10.3389/fpubh.2025.1649931 (PMC12727977; doi:10.3389/fpubh.2025.1649931)
Supplement: Supplementary file 1 [file Table_1.DOCX]

**Table SI: Constructs, Codes, and items**

| **Construct** | **Code** | **Items (5-Points Likert Scale)** |
| --- | --- | --- |
| Healthcare Providers' Knowledge | GPK | I am familiar with the concept of social prescription for psychosocial health and well-being. |
|  | GPK | I know the various non-clinical services available for psychosocial health and well-being under social prescription. |
|  | GPK | I understand the importance of social prescriptions for psychosocial health. |
|  | GPK | I am aware of the role of social prescription in psychosocial health and well-being promotion. |
|  | GPK | I received training about social prescription applications for psychosocial health and well-being. |
| Healthcare Providers Attitude | GPA | Social prescription is an essential part of a comprehensive approach to healthcare in the primary setting. |
|  | GPA | I am in favor of implementing social prescription in my clinical practice. |
|  | GPA | Social prescription improves the psychosocial health and well-being of my patients. |
|  | GPA | Social prescription is a valuable addition to the traditional biomedical model for psychosocial health and well-being. |
|  | GPA | I am open to integrating non-clinical practices into my patient care under social prescription for psychosocial health and well-being. |
| Perceived Effectiveness Social Prescription | GPPESP | Social prescription is a comprehensive approach to psychosocial health and well-being. |
|  | GPPESP | Social prescription is a cost-effective way to improve patient psychosocial health and well-being outcomes. |
|  | GPPESP | Social prescription should be a part of every healthcare provider's patient treatment plan for psychosocial health and well-being. |
|  | GPPESP | Social prescription is a valuable tool for healthcare providers in the primary care setting. |
|  | GPPESP | Social prescription is well-supported by evidence and research related to psychosocial health and well-being. |
|  | GPPESP | I feel confident in my ability to implement social prescription in my practice. |
|  | GPPESP | Social prescription is well-received by patients. |
|  | GPPESP | Social prescription is cost-effective compared to other healthcare interventions. |
|  | GPPESP | Positively impacts patients' overall psychosocial health and well-being. |
|  | GPPESP | Social prescription should be more widely adopted in healthcare practices. |
| Arts on Social Prescription | AoSP | I believe arts can be an effective social prescription for mental health and psychosocial well-being. |
|  | AoSP | Incorporating the arts into healthcare can positively impact a patient's mental health. |
|  | AoSP | I believe patients are receptive to using arts to treat their mental health. |
|  | AoSP | I think that arts-based social prescription is cost-effective compared to other mental health interventions. |
|  | AoSP | Incorporating arts into healthcare as a social prescription should be more widely adopted. |
| Nature on Social Prescription | NoSP | How strongly do you believe spending time in nature can help improve mental health and well-being? |
|  | NoSP | How confident are you in the effectiveness of nature as a social prescription for your patients with mental health conditions? |
|  | NoSP | I believe integrating nature-based activities into my patient's treatment plans would positively impact their mental health and psychosocial well-being. |
|  | NoSP | The benefits of "nature as social prescription" should be included in mental health treatment plans. |
|  | NoSP | I have prescribed "nature as social prescription" to my patients and have seen positive results. |
| Physical Activity on Social Prescription | PAoSP | I believe that physical activity should be prescribed as a form of treatment for mental health and well-being. |
|  | PAoSP | I think that physical activity is a good way for patients to improve their mental health and well-being. |
|  | PAoSP | I believe that healthcare providers should encourage their patients to participate in physical activity for their mental health. |
|  | PAoSP | I think that physical activity can be a good way for patients to reduce stress and anxiety. |
|  | PAoSP | I think that physical activity can provide a sense of accomplishment and boost self-esteem, which is vital for mental health. |
